# Intraocular pressure and aqueous humor flow during a euglycemic-hyperinsulinemic clamp in patients with type 1 diabetes and microvascular complications

**DOI:** 10.1186/1471-2415-10-19

**Published:** 2010-06-23

**Authors:** James T Lane, LuAnn Larson, Shan Fan, Julie A Stoner, Eyal Margalit, Carol B Toris

**Affiliations:** 1 Department of Internal Medicine, University of Nebraska Medical Center, Omaha, NE USA; 2 Department of Ophthalmology, University of Nebraska Medical Center, Omaha, NE 68198-5840, USA; 3 Department of Biostatistics and Epidemiology, University of Oklahoma Health Science Center, Oklahoma City, OK USA

## Abstract

**Background:**

Microvascular complications, including retinopathy and nephropathy are seen with type 1 diabetes. It is unknown whether functional changes in aqueous humor flow or intraocular pressure (IOP) develop in parallel with these complications. This study was designed to test the hypothesis that clinical markers of microvascular complications coexist with the alteration in aqueous humor flow and IOP.

**Methods:**

Ten patients with type 1 diabetes and ten healthy age- and weight-matched controls were studied. Aqueous flow was measured by fluorophotometry during a hyperinsulinemic-euglycemic clamp (insulin 2 mU/kg/min). Intraocular pressure was measured by tonometry at -10, 90 and 240 minutes from the start of the clamp, and outflow facility was measured by tonography at 240 minutes.

**Results:**

During conditions of identical glucose and insulin concentrations, mean aqueous flow was lower by 0.58 μl/min in the diabetes group compared to controls (2.58 ± 0.65 versus 3.16 ± 0.66 μl/min, respectively, mean ± SD, p = 0.07) but statistical significance was not reached. Before the clamp, IOP was higher in the diabetes group (22.6 ± 3.0 mm Hg) than in the control group (19.3 ± 1.8 mm Hg, p = 0.01) but at 90 minutes into the clamp, and for the remainder of the study, IOP was reduced in the diabetes group to the level of the control group. Ocular pulse amplitude and outflow facility were not different between groups. Systolic blood pressure was significantly higher in the diabetes group, but diastolic and mean arterial pressures were not different.

**Conclusions:**

We conclude that compared to healthy participants, patients with type 1 diabetes having microalbuminuria and retinopathy have higher IOPs that are normalized by hyperinsulinemia. During the clamp, a reduction in aqueous flow was not statistically significant.

## Background

Maintenance of intraocular pressure (IOP) within a narrow range is essential for the normal health of the eye. The IOP is kept within these limits by the production rate of aqueous humor into the posterior chamber of the eye and its drainage out of the anterior chamber angle. The production rate can be assessed by measuring the aqueous flow rate from the posterior chamber into the anterior chamber. In addition to contributing to the maintenance of IOP, the flow of aqueous humor delivers nourishment to and removes waste products from the avascular structures within the anterior segment [1]. Thus, adequate aqueous flow is crucial for the health of the eye. Alterations in aqueous flow have been described in humans with diabetes, and it is thought that this contributes to diabetes-related changes in the eye. It is unclear whether the alterations have functional consequences or reflect major pathophysiologic processes associated with diabetes-related eye disease [2-4].

We previously reported that aqueous flow was decreased by 15% in patients with type 1 diabetes without evidence of microvascular complications, compared to healthy control participants [3]. Measurements were made under rigorous conditions utilizing a hyperinsulinemic-euglycemic clamp. Such conditions were necessary to control insulin and glucose concentrations in both study groups during the measurements, as these factors could impact vascular blood flow, an important determinant of aqueous flow [5-7]. If not carefully controlled, insulin and glucose potentially could affect aqueous flow and mask or mute effects due to the diabetes. Other published studies did not control for the levels of insulin and glucose during the assessment of aqueous flow [2,4,6] making it difficult to separate the disease effect from the effect of insulin and glucose.

Severity of diabetic retinopathy is related to length and magnitude of exposure to hyperglycemia [8-10]. Diabetic retinopathy appears to exist concurrently with microvascular abnormalities in the kidney and signals diabetic nephropathy [11]. If a decrease in aqueous flow accompanies diabetic retinopathy, and if these changes are related by common pathophysiologic mechanisms, it is hypothesized that patients with evidence of retinopathy will have decreased aqueous flow that could impact IOP. The relationship between rate of aqueous flow and the severity of complications in the eyes of patients with diabetes is not known. This study examines aqueous flow under conditions of hyperinsulinemia and euglycemia in eyes of patients with type 1 diabetes with evidence of retinopathy and microalbuminuria and eyes of healthy non-diabetic age-matched controls. This differs from our previous study that measured aqueous flow in patients with type 1 diabetes but without microvascular complications [3]. Accompanying the measurement of aqueous flow were assessments of other parameters that may be affected by the disease, including IOP, outflow facility, and blood pressure.

## Methods

Ten patients with type 1 diabetes from the diabetes clinics at the University of Nebraska Medical Center and ten healthy, age-matched non-diabetic control participants from the general population were enrolled in this one-day, cross-sectional study. All studies were performed in the Clinical Research Center at the University of Nebraska Medical Center. The protocol was approved by the Institutional Review Board at the University of Nebraska Medical Center.

Inclusion criteria for patients with type 1 diabetes were a diagnosis of type 1 diabetes by ADA criteria [12] for at least 10 years and the presence of both microalbuminuria and retinopathy. Microalbuminuria was defined as ≥ 30 mcg/mg creatinine on a random urine specimen at screening, or treatment with an angiotensin converting enzyme (ACE) inhibitor or angiotensin receptor antagonist (ARB) for the presence of a previously elevated albumin excretion [13]. In all patients on ACE inhibitors or ARBs, the medical record substantiated a previously elevated albumin excretion rate of > 30 mcg/mg/cr on more than one occasion. Retinopathy was identified by a single ophthalmologist (EM) using the Early Treatment Diabetic Retinopathy Study Final Scale of Diabetic Retinopathy Severity for Individual Eyes [14]. Patients with diabetes could not have an HgbA1c > 12%, history of laser treatment to the retina, or an insulin dose > 100 units per day.

Exclusions for both groups were coronary artery disease, heart failure, cancer, AIDS, obstructive lung disease, or other chronic major medical problems, body mass index (BMI) > 30 mg/m^2^, anemia (hemoglobin < 11.5 g/dl for women and < 13.0 g/dl for men), serum creatinine > 2.0 mg/dl, a history of cigarette smoking within the previous three months, a history of alcohol intake > 2 ounces per day on a chronic basis, pregnancy, a history of transplantation, cornea defects, hypersensitivity to fluorescein, an ocular infection or inflammation or use of ocular drugs within the previous three months, a history of ocular surgery, or any medication that could affect IOP. Healthy participants could have no evidence of diabetes at screening and all participants were at least 19 years of age.

After informed consent was obtained, participants underwent a medical history, physical examination, screening laboratory tests, and a screening eye examination. Screening laboratory tests included hemoglobin, fasting plasma glucose, serum creatinine, and a random urine for microalbumin/creatinine ratio. Screening eye exams included visual acuity, pachymetry, tonometry, a slit-lamp examination, and a dilated fundus examination for retinopathy grading.

Three days prior to their clamp study, patients with type 1 diabetes were changed to NPH insulin for their basal insulin. On the night prior to the study, patients with type 1 diabetes were admitted to the hospital to receive intravenous regular insulin designed to maintain plasma glucose between 70-140 mg/dl. This prevented large fluctuations in plasma glucose levels for twelve hours before measurements commenced. The last dose of NPH insulin was given on the morning before admission. At 2:00 AM, all participants received 8 drops of fluorescein 2% in each eye with a five-minute interval between drops.

On the day of the study, all participants were admitted to the Clinical Research Center at the University of Nebraska Medical Center following an overnight fast. Both groups were studied using a hyperinsulinemic-euglycemic glucose clamp [15]. Insulin and glucose were infused at controlled rates using Harvard pumps (Model 22 Multi; Harvard Bioscience, Holliston, MA). Insulin was infused at a constant rate of 2 mU/kg/min (Humulin^®^, Eli Lilly, Co. Indianapolis, IN), and glucose was variably infused to maintain plasma glucose at 90 mg/dl. The hand used to sample arterialized venous blood was placed in a hot box maintained at 60°C for the duration of the study. Potassium phosphate (30 meq in 250 cc saline 0.45%) was infused at 30 ml/hr during the clamp procedure.

Aqueous flow was measured using the intracameral fluorescein dilution technique, as quantified by a Fluorotron Master fluorophotometer (Ocumetrics, Palo Alto, CA) [16]. Anterior chamber volume was calculated from anterior chamber depth and corneal thickness measurements collected during screening. The amount of fluorescein in the cornea and anterior chamber was measured at -10 and +90 minutes from the start of the clamp and every 45 minutes thereafter for 4.5 hours. The clamp procedure commenced at zero minutes. Aqueous flow was determined as described previously [16,17].

Intraocular pressure was measured at -10 minutes, +90 minutes, and +4.5 hours using a pneumatonometer (Reichert Ophthalmic Instruments, Depew, NY). Two readings of each eye were made and averaged. Ocular pulse amplitude was obtained from the pressure wave. A two-minute tonography measurement (tonography setting on the Classic 30 pneumatonometer, Reichert Ophthalmic Instruments, Depew, NY) was performed on each eye at +4.5 hours for determination of outflow facility [18].

Analyses of hemoglobin A1c, serum creatinine, and urine albumin and creatinine were performed in The Nebraska Medical Center's Clinical Laboratory using standard methods. Hemoglobin A1c was measured using high-pressure liquid chromatography (normal range: 4.0-6.0%). Urine albumin was measured by nephelometry. During the clamp procedure, plasma glucose was measured every five minutes using a glucose oxidase method (Glucose Analyzer II; Beckman, Fullerton, CA). Plasma insulin levels were measured by radioimmunoassay (Linco Research, Inc, St. Louis, MO) with 0.2% cross-reactivity with proinsulin and a sensitivity of 12 pmol/l. The inter-assay and intra-assay coefficients of variation were 7.9% and 2.5%, respectively.

The glucose infusion rate during the final 30 minutes of the clamp was used to compare insulin sensitivity between groups [19]. Mean arterial pressure (MAP) was calculated using the formula: MAP = [SBP + (2 × DBP)]/3, where SBP is systolic blood pressure and DBP is diastolic blood pressure.

Baseline characteristics were compared between control and diabetic participants using a non-parametric Wilcoxon rank sum test for continuous measures or a Fisher's exact test for categorical measures. Changes in the eye-level outcome measures over time were compared between the normal and diabetic participants using a nested mixed effects ANOVA model. The ANOVA model was fit using right and left eye data (combined) where the correlation within a participant was accounted for using a random participant effect nested within diabetes status. The model also included a fixed diabetes status term, a fixed time term, and the interaction between time and diabetes status. Natural log transformations of the response were modeled when necessary to meet ANOVA modeling assumptions. Pair-wise comparisons among time points were made using a Tukey's adjustment. Given the limited sample sizes, a simplified sensitivity analysis approach was used where changes over time were calculated using the mean of right and left eye data and comparisons made between diabetic and non-diabetic participants using a non-parametric Wilcoxon rank sum test. Results of this simplified approach were similar to results from the mixed effect ANOVA modeling approach and are therefore not presented. Descriptive statistics are based on the average of right and left eye measurements and all descriptive results are expressed as means ± standard deviation (SD).

Based on our previous study [3], we calculated that a sample size of 10 participants per group would be needed to detect a difference between a mean aqueous flow value of 2.4 μl/min for the diabetes group and 3.2 μl/min for the control group with 80% power assuming a common standard deviation of 0.7 ml/min, a 2-sided 0.05 alpha level and a correlation coefficient of 0.7 between the right and left eyes of each participant [20].

## Results

A total of 20 participants (10 patients with type 1 diabetes and 10 healthy controls) were studied. Participants were similar in age, gender, ethnicity, body mass index, diastolic blood pressure, mean arterial blood pressure, and serum creatinine (Table 1). Patients with type 1 diabetes had higher systolic blood pressure (119 ± 14 versus 105 ± 9 mm Hg, p = 0.04), although mean arterial pressure was not different between groups. As expected, the patients with diabetes had higher HbA1c levels, compared to healthy controls. The patients had type 1 diabetes for a mean of 25 years.

**Table 1:** Participant Characteristics

Characteristics	Groups		P-Value*
	Type 1 Diabetes (n = 10)	Controls (n = 10)	

Age (years)	35.2 (12.5)	34.9 (9.4)	> 0.9

Female	4 (40%)	5 (50%)	> 0.9

Ethnicity			> 0.9
Caucasian	8 (80%)	8 (80%)	
African American	2 (20%)	1 (10%)	
Asian American		1 (10%)	

Height (cm)	170.4 (10.9)	171.2 (10.7)	0.7
Weight (kg)	76.9 (15.5)	83.4 (35.3)	0.9
BMI (kg/m^2^)	26.3 (4.3)	24.6 (2.7)	0.8

Duration of diabetes (yrs)	25.4 (8.4)	N/A	

Systolic BP (mm Hg)	119 (14)	105(9)	0.04
Diastolic BP (mm Hg)	71 (10)	65 (5)	0.2
Mean arterial BP (mm Hg)	87 (11)	79 (5)	0.1

HbA1c (%)	9.1 (2.2)	5.1 (0.3)	0.001

S creatinine (mg/dl)	1.0 (0.3)	1.0 (0.1)	0.5

Urine microalbumin (μg/mg cr)	367.4 (668.7) Median = 16.9	4.32 (1.5) Median = 3.9	0.005

Results are mean ± (SD), unless otherwise noted

*P value by Wilcoxon rank sum test or Fisher's exact test

N/A, not applicable

By design, the patients with diabetes had higher albumin excretion rates, as determined by a random urine for albumin to creatinine ratio. The mean albumin excretion rate was in the macroalbumin range, but the median value of 16.9 mcg/mg creatinine was not.

Retinopathy was present in all 10 patients with diabetes. One participant had retinopathy grade 1 in the right eye and no retinopathy in the left eye. For this patient, only the eye with retinopathy was used in the data analysis. Seven participants had retinopathy grades of 1 for both eyes. One participant had retinopathy grade 2 in the right eye and 3 in the left. One participant had retinopathy grade 4 in the right eye and 3 in the left. All control participants had no retinopathy.

**Figure 1:**
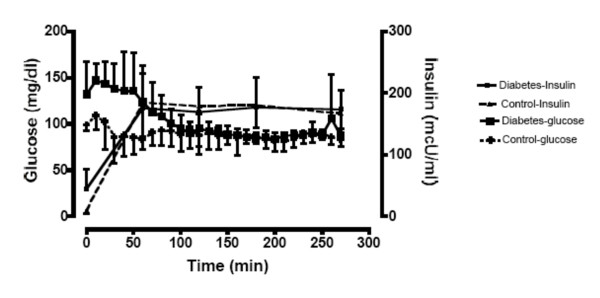
**Glucose and insulin concentrations (mean ± SD) over time during the hyperinsulinemic-euglycemic glucose clamp in patients with type 1 diabetes and age-matched healthy controls (n = 10)**.

The insulin and glucose levels during the euglycemic-hyperinsulinemic glucose clamp are summarized in Figure 1. At baseline, mean total insulin levels were significantly greater for patients with diabetes, compared to controls (p < 0.001). Within one hour, insulin concentrations were similar between groups and remained that way for the remaining 3.5 hours. Insulin concentrations during the last hour of the clamp were similar (174.1 ± 30.6 mU/ml in patients versus 166.8 ± 33.9 mU/ml in controls, p = 0.6). Glucose concentrations at the beginning of the clamp were higher in patients with type 1 diabetes, compared to controls. By 80 minutes into the clamp the glucose levels were similar between groups and remained similar for the rest of the clamp. The whole body glucose infusion rate during the last 30 minutes of the clamp, an indication of insulin sensitivity, was lower in patients with type 1 diabetes, compared to controls (7.6 ± 3.7, versus 11.7 ± 2.9 mg/kg/min, respectively, p = 0.01).

The mean aqueous flow rate in patients with type 1 diabetes was decreased compared to healthy controls, although this decrease did not reach statistical significance (p = 0.07). The baseline IOP was significantly higher in the patients with diabetes compared to controls. Using a mixed effects ANOVA model, the IOP in the diabetes group decreased over time, whereas the IOP in the control group remained unchanged (p = 0.03). Within the diabetes group, the IOP decrease over time occurred between +0 and +90 minutes with no further change between +90 and +240 minutes. Mean tonographic outflow facility at 240 minutes into the clamp was similar between the two groups. A summary of these results are found in Table 2.

**Table 2:** Aqueous flow, outflow facility and IOP in patients with type 1 diabetes and healthy age-matched controls

Measurement	Type 1 Diabetes (n = 10)	Controls (n = 10)	P-Value*
Aqueous flow (μl/min)	2.58 ± 0.65	3.16 ± 0.66	0.07

IOP, +0 min (mmHg)	22.6 ± 3.0	19.3 ± 1.8	0.01

IOP, +90 min (mmHg)	20.1 ± 3.6	19.3 ± 2.5	0.6

IOP, +240 min (mmHg)	21.1 ± 3.1	19.1 ± 1.9	0.1

Outflow facility (μl/min/mmHg)	0.32 ± 0.10	0.32 ± 0.09	0.98

Results are mean ± (SD) unless otherwise indicated.

IOP, intraocular pressure

IOP, 0 is the measurement just before the glucose clamp began.

IOP, +90 is the IOP at ninety minutes into the clamp

IOP, +240 is the IOP at the end of the clamp procedure.

*comparing type 1 diabetes with controls by ANOVA

## Discussion

In the present study, the 19% decrease in aqueous flow in the diabetes group with microvascular complications compared to the control group, was similar in magnitude to our previous study of patients with type 1 diabetes and no complications (15%) [3]. A finding in the earlier study that aqueous flow was slowed, despite no diabetic complications, led us to hypothesize that aqueous flow is slowed even further in the presence of complications. This did not prove to be the case. It is possible that the complications were sufficiently minor and the standard diabetes treatments sufficiently effective that aqueous flow was not reduced further in the presence of mild microvascular complications. By design, our participants exhibited only mild evidence of microvascular complications in the retina (retinopathy grade of 1 in seven of the ten patients) and kidney (microalbunimuria in all patients).

Another consideration for the lack of a greater decline in aqueous flow than expected is the use of ACE inhibitors in all patients with type 1 diabetes in this study. This is standard care for patients with microalbuminuria. These drugs are known to prevent thickening of the glomerular basement membrane in diabetic rats [21]. ACE inhibitors might protect the basement membrane of the ciliary body in a similar manner and protect against diabetes-related changes in aqueous production. A study in normal rabbits reported that topical ACE inhibitors reduced IOP and aqueous flow [22]. Unlike the rabbit study, our study did not find reduced IOP in the diabetes group. In fact, IOP before the clamp procedure was higher in the diabetes group, compared to the control group, suggesting that the ACE inhibitors were not prescribed at a dose sufficient to maintain IOP within normal levels. Hence, the aqueous flow effect was more likely due to the diabetes than the ACE inhibitor.

It remains to be seen whether aqueous flow decreases further with advanced diabetic retinopathy. This would be a difficult study to design as most patients with advanced retinopathy are treated with laser photocoagulation which can affect the measurement of aqueous flow. Laser treatments to the eye may cause chronic low-grade inflammation, and the flare and cells in the anterior chamber might interfere with the measurement of intracameral fluorescein used in the determination of aqueous flow [23].

Previous studies [2,4] have reported decreased aqueous flow in patients with diabetes who did not undergo a hyperinsulinemic-euglycemic clamp. Compared to healthy controls, patients with type 1 diabetes are more insulin resistant and are exposed to higher insulin concentrations [3]. As insulin and glucose levels are known to have an effect on blood flow in tissues [5-7] and blood flow is a determinant of aqueous flow [1,24], we performed a hyperinsulinemic-euglycemic glucose clamp to obtain similar insulin and glucose levels between groups during the time of the aqueous flow measurements. The insulin levels were purposefully kept in the upper range of the dose-response curve for insulin to suppress hepatic glucose production and allow the glucose infusion rate to equal total body glucose utilization, an indication of insulin sensitivity.

Our study has several limitations of note. First, due to the complexity of the measurement methods and the difficulty in finding appropriate and willing participants, it is a small study with limited power. Prior to the study start a power analysis indicated that the numbers enrolled would be sufficient to detect expected statistically significant differences in aqueous flow. However, this did not happen. Also, observed effect sizes for secondary endpoints, including intraocular pressure and outflow, were smaller than the assumed standardized effect size for aqueous flow, resulting in less than 80% power to address these endpoints. Second, the design of the study only allows us to examine acute ocular changes during specific conditions of insulin and glucose concentrations. Although patients with diabetes were selected based on their burden of microvascular complications, there was no control for long-term exposure to hyperglycemia prior to the study start. Finally, the insulin concentrations during the clamp studies were higher than those experienced during daily living. Results may have been different under different experimental conditions.

Two important findings in our study were that systolic pressure and IOP were significantly greater in patients with type 1 diabetes compared to healthy controls. This agrees with several recent epidemiologic studies that report an association between systolic pressure and elevated IOP [25-28] and association between diabetes and elevated IOP [26,29,30]. It has been reported recently that diabetic patients have lower corneal hysteresis than healthy controls, which could explain, in part, the higher IOPs in this group [31-34]. Further research in this area would be of interest. Diabetes is not always associated with elevated IOP [3,35], but if it is found, it could be explained in part by higher insulin resistance [36] and chronic hyperglycemia [37]. Since insulin acts as a vasodilator through nitric oxide in the eye, there may be some common effect between insulin and IOP [38]. It has been shown that nitric oxide decreases IOP acutely by increasing trabecular outflow facility [39,40]. It has been postulated that IOP falls acutely in response to nitric oxide-induced contraction of cell volume through such mediators as soluble guanylate cyclase, protein kinase G, and the large-conductance Ca^2+ ^-activated K^+ ^channel [41]. In fact, these regulatory elements may associate insulin with changes in outflow facility. Why the patients with type 1 diabetes had a greater response to acute insulin, compared to controls, may be because of the presence of generalized insulin resistance.

The rapid IOP fall in patients with type 1 diabetes in response to intravenous insulin is a novel finding. This is the period of equilibration of insulin in the peripheral tissues. The IOP reduction during this time may have been in response to the increase in circulating insulin levels, which may have had the effect of overcoming any insulin resistance, especially on local blood flow. After the first ninety minutes and for the duration of the study, there was no difference in IOP between groups. Patients with relatively well-controlled type 1 diabetes in which retinal blood flow was measured by laser Doppler flowmetry with minimal background retinopathy were similar to control participants [42]. Most studies characterizing the effect of insulin on IOP and ocular fluid dynamics have reported averages over many hours to days. Rapid fluctuations in glucose and insulin concentrations apparently can affect IOP acutely. Rapid reductions in blood flow sufficient to slow aqueous flow and hence, reduce IOP, could explain these findings. Even with rigorous glucose control, patients with type 1 diabetes often experience insulin and glucose fluctuations that apparently affect IOP. The implications for normal physiology and disease as they relate to the frequency and amplitude of IOP changes over time could be significant.

An additional finding of interest in the current study is the normal IOP in the diabetes group during the clamp despite the presence of reduced aqueous flow. If all other parameters of aqueous humor dynamics remained unchanged, a reduction in aqueous flow alone should reduce IOP. Since this did not happen, other changes in aqueous humor dynamics may be occurring concurrent to the slowed aqueous flow. Such changes could include an increase in episcleral venous pressure, reduction in trabecular outflow facility, or reduction in uveoscleral outflow. Outflow facility was not different between groups when measured by two-minute tonography in our study and another [4]. A reduction in episcleral venous pressure is possible, reflecting acute effects on blood flow. Ocular pulse amplitude was not changed by diabetes or insulin in our study which agrees with another study of patients with diabetes who were not subjected to the glucose clamp [43]. This implies that choroidal perfusion and ocular blood flow remain normal in these patients. A reduction in uveoscleral outflow is the likely explanation for the normal IOP despite a reduction in aqueous flow. This possibility is not without some evidence. A number of tissues demonstrate increased matrix deposition and basement membrane thickening in response to short- and long-term hyperglycemia [44-47]. Increased matrix deposition has been identified in the ciliary muscle (site of uveoscleral outflow) of aging rhesus monkeys when compared to young monkeys [48,49]. Uveoscleral outflow is reduced with age in humans [50] which may be related to excessive buildup of matrix material over decades. Therefore, in diabetes, there could be an association between the rate of uveoscleral outflow and matrix deposition in diabetes.

## Conclusions

Patients with type 1 diabetes, having microalbuminuria and diabetic retinopathy, had IOPs and baseline systolic blood pressures that were higher than healthy controls. During the performance of a hyperinsulinemic-euglycemic glucose clamp when insulin and glucose levels were similar between groups, IOP was reduced to control levels. Aqueous flow rate over the entire clamp period trended lower in the diabetes group but was not statistically significant, while outflow facility remained unchanged. Further studies under controlled conditions in more advanced diabetic retinopathy are required to determine if major alterations in aqueous flow are manifest.

## Competing interests

The authors declare that they have no competing interests.

## Authors' contributions

JL designed the study, recruited the patients, performed the euglycemic -hyperinsulinemic clamps for each patient, reviewed the data, and wrote the manuscript. LL supervised the care of patients and collected data as nurse manager in the Clinical Research Center. SF performed IOP and flurophotometric measurements on the patients. JS was the chief statistician. EM performed all screening eye exams and retinopathy gradings. CT helped design the study, supervised the collection of all eye data, performed data analysis, and helped with the preparation of the manuscript. All authors read and approved the final manuscript.

## Pre-publication history

The pre-publication history for this paper can be accessed here:

http://www.biomedcentral.com/1471-2415/10/19/prepub
